# CooC11 and CooC7: the development and validation of age appropriate children’s perceived cooking competence measures

**DOI:** 10.1186/s12966-021-01089-9

**Published:** 2021-01-30

**Authors:** Moira Dean, Johann Issartel, Tony Benson, Amanda McCloat, Elaine Mooney, Claire McKernan, Laura Dunne, Sarah F. Brennan, Sarah E. Moore, Danielle McCarthy, Jayne V. Woodside, Fiona Lavelle

**Affiliations:** 1 Institute for Global Food Security, School of Biological Sciences, Queen’s University Belfast, Belfast, UK; 2 School of Health Sciences, Faculty of Health and Medicine, The University of Newcastle, Callaghan, NSW 2308 Australia; 3 Multisensory Motor Learning Lab, Dublin City University, Dublin, Ireland; 4 Department of Home Economics, St. Angela’s College, Sligo (National University of Ireland Galway), Sligo, Ireland; 5 Centre for Evidence and Social Innovation, Queen’s University Belfast, Belfast, UK; 6 Centre for Public Health, Queen’s University Belfast, Belfast, UK

**Keywords:** Measure, Development, Validation, Cooking, Children, Intervention, Assessment, Nutrition, Motor skills, Competence

## Abstract

**Background:**

Learning cooking skills during childhood and adolescence is associated with positive dietary outcomes in adulthood as well as being tracked from adolescence to adulthood. In addition studies have found that perceived competence to be a greater motivator to perform a behaviour than actual competence. However, a lack of validated tools that effectively measure behavioural and dietary changes including cooking confidence in children is a limitation. Therefore, this research aimed to develop and validate age-appropriate perceived cooking competence measures for younger and older primary school aged children.

**Methods:**

Two measures of perceived **Coo**king **C**ompetence (**CooC11** and **CooC7**) for older (8–12 years) and younger (6–7 years) children were developed from a critical evaluation of publically available recommendations and expert consultation. The cooking skills within the measures were illustrated by a graphic designer in consultation with a chef and reviewed in an iterative manner by the research team. The measures were piloted for clarity, ease of use and initial face validity. Multiple studies were used for both **CooC11** and **CooC7** to establish psychometric properties of the measures, temporal stability, internal consistency reliability, construct validity, as well as responsiveness to change for **CooC11**. Analysis included Exploratory Factor Analysis, Confirmatory Factor Analysis, Intraclass Correlation Coefficients, Pearson’s Correlations, ANOVAs and Cronbach’s Alphas.

**Results:**

Both measures had high levels of face validity and received positive user feedback. Two factors were shown in both measures with the measures showing excellent temporal stability (ICC > 0.9) and good internal consistency (Cronbach’s Alphas > 0.7). Both measures showed initial discriminant validity, with significant differences (*P*< 0.001) between those who reported assisting their parents with dinner preparation and those who did not. Additionally, **CooC11** was significantly correlated with an adult cooking measure and had a significant responsiveness to change (*P*< 0.01).

**Conclusions:**

The **CooC11** and **CooC7** are the first validated age-appropriate measures for assessing children’s perceived **Coo**king **C**ompetence for ages 8–12 and 6–7 years respectively. They can be used to evaluate the efficacy of children’s cooking intervention studies or school nutrition education programmes.

## Background

Diet quality has been associated with a number of health outcomes including all-cause mortality, cardiovascular disease risk, cancer risk, and obesity, as well as non-health related outcomes such as academic performance [1–4]. Due to the beneficial associations of a higher diet quality, nutrition education programmes are being used for changing children’s dietary patterns and intakes to increase healthy eating practices [5, 6]. Within this area, cooking interventions have been highlighted as a promising method for changing children’s food-related attitudes, preferences and behaviours [7, 8]. Research shows that learning cooking skills at younger ages is associated with positive dietary outcomes in adulthood and these skills track from adolescence to adulthood [9, 10]. Additionally, consumption of meals prepared in the home environment, which require cooking skills, has been associated with a normal BMI and body fat percentage [11]. However, a lack of validated measurement tools that effectively measure behavioural and dietary competencies in children is a limitation not only in cooking research [8] but is also an issue in the wider nutrition area [12, 13].

In recent years, a small number of child-orientated measures have been developed in the nutrition area covering topics such as Nutrition and Food Label Literacy [14, 15]. Yet, parental perspectives of child behaviours are still often used as a measure which can lead to bias [16]. Within children’s cooking interventions, while some effort has been made to develop a validated measure [17], this measure tends to focus on broader concepts such as preparing a snack with fruit or vegetables, following a recipe or making a salad etc. and are not specific to measuring individual cooking skills such as chopping, stirring or peeling.

In both adults and children, increased confidence and self-efficacy (situation specific self-confidence [18]) are key contributors to engaging in cooking practices and repeating the behaviour [19–23]. The self-efficacy people have for a specific task, in this case cooking, contributes to the individual’s perceived competence (‘the perception a person has concerning his or her abilities’ [24]). Studies have found perceived competence to be a greater motivator to perform a behaviour than actual competence [25]. This has been extensively studied in the area of physical activity, where children with higher levels of perceived competence participated in a greater amount of physical activity [26]. Additionally, higher levels of perceived competence at younger ages predicted higher levels of perceived competence and physical activity at older ages [27]. Furthermore, children with low levels of perceived competence, even with high actual competence, were shown to have lower levels of motivation for physical activity than children with high levels of perceived competence (with or without matching levels of actual competence) [28]. Therefore, being able to measure perceived competence effectively is essential for understanding behaviour change and for evaluating successful interventions. While measuring perceived competence in motor skills exist [29, 30] currently there is no equivalent perceived competence measurement tool in the area of cooking, which is also a learned and modifiable behaviour.

The developmental differences between children and adults require consideration when conducting research with children [31]. Therefore, when developing measures appropriate to children, the developmental stages and capabilities of the child, such as their motor skill development, must be taken into consideration for the content of the measure, as well as a child’s attention span, the format of the measure, the validity and reliability of the responses and the clarity of the language [31, 32]. In addition, recommendations from the literature [31, 33] suggest that, when children are involved, research methods using visual and/or game-like measurements are preferred. These methods are more engaging to children and are similar in formats to teaching methods used at school [31, 33]. These strategies have been implemented in the children’s perceived motor skills competence measure [29, 30]. They have shown to be effective in the measurement of perceived motor skills competence, globally [34–38]. However, while using these methods may be engaging for the children, it must also obtain relevant data [31]. Therefore, it is necessary to develop appropriate and relevant items within the measure. The specific cooking skills within the measure must be relevant to the children’s developmental capacity to ensure that children are rating their perceived competence on items that they are able to achieve (i.e. appropriate for their age) [39]. Therefore, this research aimed to develop and validate age-appropriate perceived cooking competence measures for children, through the assessment of the measures’ face validity, psychometric properties, construct validity and reliability (both internal consistency and temporal stability).

## Methods

A number of steps were undertaken in the development and validation processes of the two age-appropriate children’s cooking competence measures. Items were selected for inclusion for the content of the measures from a review of children’s cooking recommendations that were mapped to their age-appropriate developmental skills [39], and were reviewed by an expert panel. The measure was designed in line with a published perceived motor competence measure [30] and characters performing the cooking skills were illustrated by a graphic designer. Next initial face validity was established by the research team and a primary school teacher and the measures were piloted. The psychometric properties of the measures were assessed using Exploratory Factor Analysis (EFA) and Confirmatory Factor Analysis (CFA) and face validity of the final structures of the two measures were established. Following this, construct validity, convergent and discriminant, and internal consistency reliability were assessed. Temporal stability reliability of both measures was then examined. Finally, for the older measure, responsiveness to change was established. The details of this process can be found in the following sections.

### Item selection

The cooking skills that children should be learning at different ages were obtained through a critical evaluation of publically available children’s recommendations and the addition of new recommendations based on children’s developmental skills [39]. From this review, for the two **Coo**king **C**ompetence measures, 14 cooking skills for 8–12 year olds (**CooC11**) and 10 skills for 6–7 year olds (**CooC7**) were identified as being frequently occurring and culturally neutral, i.e. a cooking skill was not specific to one culture. The items were selected to ensure they were both developmentally appropriate and relevant [31]. An expert panel including an educational researcher, a primary school teacher, an early year’s educator, a movement scientist and two Home Economists, with a minimum of 10 years’ experience in their respective fields, reviewed the selected skills for age appropriateness and level of difficulty. An age range was proposed for each skill and skills were then ranked in order of difficulty from easiest to hardest, see Table 1.

**Table 1:** Cooking skills identified for younger and older children

Level of difficulty	Cooking Skills	
	*Younger (ages 6–7 years)*	*Older (ages 8–12 years)*
Easiest		
	Tearing Leaves	Tearing Leaves
	Washing Vegetables	Washing Vegetables
	Stirring/Mixing ingredients	Stirring/Mixing ingredients
	Mashing	Mashing
	Measuring liquids^a^	Measuring liquids^a^
	Weighing ingredients^a^	Weighing ingredients^a^
	Chopping	Chopping
	Grating	Using a blender^c^
	Peeling	Grating
	Using a tin opener^b^	Peeling
		Using a microwave^d^
		Using a tin opener
		Using the oven^d^
Most difficult		Using the stove/hob^e^

^a^ – measuring liquids and weighing were separated in the measure; ^b^ - tin opener was placed in the younger age before the expert panel review moved it into the 9+ age category; ^c^ – blender replaced mixer as mixer was more associated with baking as opposed to cooking; ^d^ – oven and microwave were separated in the measure; ^e^ – Using the stove/hob was added as a means of factoring in the use of a cooker/cooker top for those that may not have an oven/as a means of trying to include stirring over heat.

### Development and implementation of measure

**Fig. 1:**
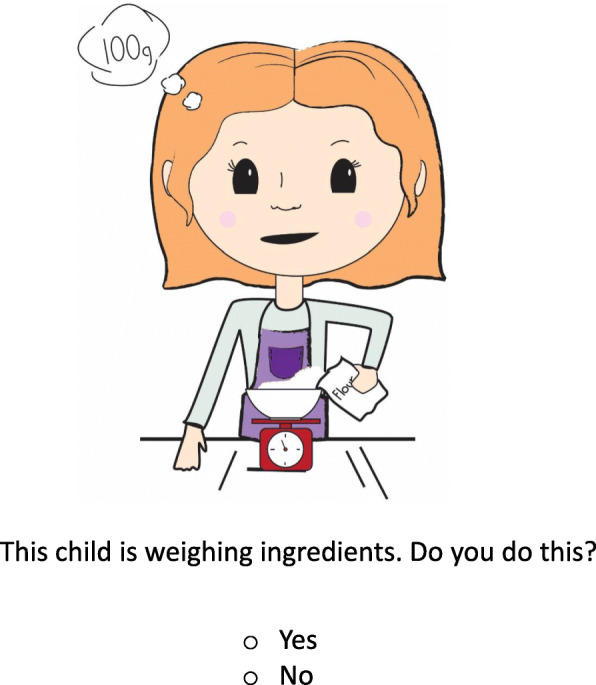
Exemplar of a cooking skill (female version)

The design of the children’s perceived cooking competence measure was based on a published perceived motor competence measure [30]. However, in the cooking skills measure the child was asked first whether they engaged in a particular cooking skill (in line with Lavelle et al. [40]), before they rated their level of competence. This aims to reduce positive illusory and social desirability biases [31]. In the measure, each skill was illustrated as a child-friendly character performing the skill. Using an iterative process, the child characters were drawn by a graphic designer in consultation with a chef and reviewed by the research team for accuracy and suitability. The illustrations provided a visual ‘cue’ to the cooking skill as some cooking terminology relating to skills may not be familiar to the children. In line with Barnett et al. [30], the child is shown an image of a child, boys are presented with images of boys performing the skills and girls are shown images of girls. This promotes a peer modelling effect, as it is argued that a child is more likely to relate to a character that is more like themselves [41, 42]. The child is asked whether they do the skill shown (see Fig. 1 as an example). If the child responds yes, then they are shown two more images of the child performing the skill, one performing it well and the other performing it poorly. The child is then asked which image represents their perceived level of competence on a five point Likert scale.

**Fig. 2:**
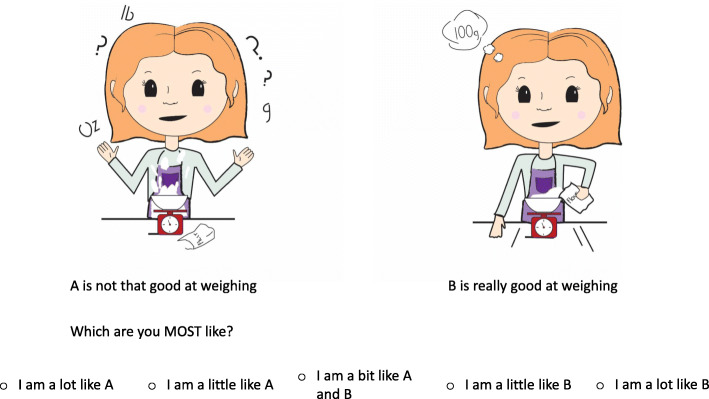
Exemplar of poor and good performance of a cooking skill presented to a child if they have indicated that they perform the skill

The five response options result in five possible levels of competence for each skill (see Fig. 2 as an example). However if the child responds that they do not perform that particular skill, they move on to the next skill.

The cooking skills are presented in ascending level of difficulty as rated by the expert panel. Additionally, the sequence of presentation of ‘good’ competence of a skill alternated in position on the page with ‘poor’ competence of a skill [29, 30]. Each child completes the measure individually. However, if a child’s literacy levels were not at a sufficient level that they could read the questions, then the researchers assisted the child by reading out the questions so that the child could complete the task.

### Piloting and initial face validity

The measures were reviewed by the research team and a primary school teacher for language, readability and literacy levels [31]. Based on the feedback minor amendments were made to the language, such as changing ‘in between A and B’ to ‘A bit like A and B,’ as it was suggested that children would interpret the original phrasing to mean physically in between the two characters. Additionally, the font size of the text was increased for the younger age group. Furthermore, the characters’ expressions were all changed to neutral, so that the children would not choose their responses based on how happy or sad they were feeling but on their perceived level of competence. Thus, the research team assessed the measures for initial face validity.

The designed measures were also piloted with a number of children of differing ages [43]. This piloting allowed the research team to assess the usability, length of time of completion, enjoyability as well as further face validity such as recognition of the skills and differentiation between the ‘good,’ and ‘poor’ performance of the skill.

Further validation assessments were undertaken for both the older age measure (**CooC11**) and the younger age measure (**CooC7**), which will be detailed in the following section. For both measures, endpoint user feedback from both the children and teachers were received. Five teachers and three teaching assistants, from the recruited samples of children, provided their perceptions around the measures including the suitability, usability and length. Each class in these samples were asked about their experiences using the measure, whether they liked this type of activity, about the characters and if there was anything they would change. Additionally, informal qualitative feedback was gathered across all samples. Prior to data analysis, where necessary, items were reversed coded so that a higher score indicated greater perceived cooking competence for all items. All analyses were conducted using IBM SPSS Statistics v25 and IBM SPSS Amos v25, with a significance level of 0.05.

### CooC11 (8–12 year olds)

#### Participants and procedure

Sample 1: Data from 469 primary school children aged 10–11 years completed baseline measurements as part of a larger study (Project Daire) [44]. For this sample, 50.32% were female. Schools from both rural and urban areas with varying socioeconomic levels were included. Data was collected in February–March 2019.

Sample 2: Children (*N*=38) between the ages of 8–9 years and 10–11 years (two year groups in the primary school system in Northern Ireland) were recruited. Children from one primary school were recruited for this study. For this sample, 52.6% were female. Children in this sample completed the measure at two time points two weeks apart in May 2019.

Sample 3: Children (*N*=32) between the ages of 10–12 years who participated in a one week cooking camp intervention in August 2019. In this sample, 78.1% were female. These children completed the measure before and after the cooking camp intervention.

#### Psychometric testing, validation and data analysis

##### Exploratory factor analysis

Sample 1 was randomly split to conduct EFA and CFA, with 269 children included in the EFA. EFA (maximum likelihood) with direct oblimin rotation was used. This oblique rotation was used as it was believed that factors would be related [45]. Sample adequacy was assessed using Kaiser-Meyer-Olkin (KMO) value [46] and Bartlett’s Test of Sphercity [47]. Factors were assessed using Eigenvalues greater than 1 [48] and a minimum of 3 items per factor [49]. Items were removed based on communalities and factor loadings.

##### Confirmatory factor analysis and face validity

The remaining 200 randomly selected children from sample 1 were used for CFA. The final model identified by the EFA was assessed as a confirmatory factor analysis with maximum-likelihood estimation, using IBM SPSS Amos v25. The following fit statistics were used to assess the model [50]:

- Chi-square (χ^2^) – A non-significant chi-square value (*p* > 0.05) which is two or three times larger than its value divided by the degrees of freedom (df) at its maximum indicates that the model can be accepted.
- Root Mean Square Error of Approximation (RMSEA) – A preferred value is 0.05 or less.
- Comparative Fit Index (CFI), Normed-Fit Index (NFI), Tucker-Lewis Index (TLI) – For these indices a value of 0.90 or greater indicates that the model can be accepted.

To establish face validity of the measure structure, five researchers in the areas of food, nutrition, health psychology, Home Economics and human movement science, reviewed the final model and factor structure. Cooking skills in each factor were assessed upon general relation in cooking as well as underlying developmental skills including fine and gross motor skills in addition to numeracy, literacy and safety considerations. All items were assessed to ensure they measured what they claimed to measure.

##### Construct validity – convergent and discriminant validity

Sample 1 was used for Construct validity. Convergent validity shows that measures are valid by identifying a relationship with an existing similar measure using correlation analysis. As there are no similar children’s measures to establish convergent validity, the cooking method section of an adult measure was used [40]. This measure has not been used previously with children due to the levels of literacy required. However, as the current sample is at the older end of the age range for the measure, the research team decided to include the measure as a means of establishing some level of convergent validity. Additionally, due to the lack of measurements available, the children were asked whether they help their parents making the dinner. It was expected that those who assist with dinner preparation would have a reported higher cooking competence. Due to a large number of children answering ‘sometimes’ (*N*=298) and a small number answering ‘always’ (*N*=35), compared with those answering ‘never’, ‘sometimes’ and ‘always’ were combined to those who ‘help with dinner.’ And due to the large difference between the two response categories, a random selection of those who responded ‘help with dinner’ were selected to compare against never. This ensured that there was a relatively equal number of participants in each group for the one-way ANOVA.

##### Internal consistency reliability

Internal consistency reliability was used to examine agreement between the items in a scale. Cronbach’s Alpha was used to assess internal consistency reliability. A value of 0.7 or higher shows good reliability [51]. Sample 1 and 2 were used to establish internal consistency of the measure.

##### Temporal stability

Sample 2 was used to assess Temporal Stability of the measure. The temporal stability of the scales was examined using the Intraclass Correlation Coefficient (ICC). This illustrates the level of agreement between item answers over time. A stronger ICC indicates greater agreement, suggesting greater temporal stability. Moderate reliability is seen with an ICC value of 0.50–0.75, good reliability is a value of 0.75–0.90, while a value of greater than 0.90 suggests excellent reliability [52].

##### Responsiveness to change

Sample 3 was used to assess the responsiveness to change of the measure, a further indication of validation [53]. This was established through investigating changes in the measure scores before and after the children receive a cooking focused intervention using T-tests.

### CooC7 (6–7 year olds)

#### Participants and procedure

Sample 4: Data from 514 primary school children aged 6–7 completed baseline measurements as part of a larger study (Project Daire) [44], are used as Sample 4. For this sample, 48.63% were female. Schools with varying socioeconomic levels and from both rural and urban areas were included. Data was collected in February–March 2019.

Sample 5: Children (*N*=13) between the ages of 6–7 years old were recruited as part of Sample 5 from the same school as sample 2. In this sample, 46.2% were female. Children in this sample completed the measure at two time points two weeks apart in May 2019.

#### Psychometric testing, validation and data analysis

The same criteria as in 2.4.2 were used for testing the **CooC7** measure. The samples used, and differences in analysis to **CooC11** are detailed below.

##### Exploratory factor analysis

Sample 4 was randomly split to conduct Exploratory Factor Analysis (EFA) and Confirmatory Factor Analysis (CFA), with 314 children included in the EFA.

##### Confirmatory factor analysis and face validity

The remaining 200 randomly selected children from Sample 4 were used for CFA. The same procedure was used for the CFA and face validity as in section 2.4.2.

##### Construct validity – discriminant validity

Sample 4 was used for construct validity. There are no similar children’s measures to establish convergent validity and the cooking method section of the adult measure [40] is above the literacy and cognitive capacity of this age group. Due to the lack of measurements available, the children were asked whether they help their parents making the dinner, with the expectation again that a higher cooking competence score would be seen in those that help with dinner preparation. The responses for the children were ‘never’, ‘sometimes’ or ‘always’. Comparisons between children in the 3 categories were conducted using an ANOVA with Scheffe post hoc analysis due to differences in numbers in the groups.

##### Internal consistency reliability and temporal stability

Sample 4 and 5 were used to establish internal consistency of the measure. Sample 5 was used to assess Temporal Stability of the **CooC7**.

### Ethical considerations

All schools partaking in the research (Samples 1, 2, 4 and 5) signed and returned a memorandum of understanding. An opt-out parental consent system was implemented. In sample 3, due to the nature of the intervention and the demand for places, an opt-in system was used. In all samples, parents were made aware that they were not obliged to allow their child to take part in the study and that they could withdraw their child at any time point up to data analysis without reason or consequence. Additionally, the children were made aware that they did not have to take part. The research was conducted in accordance with the Declaration of Helsinki. Ethical approval was received from The School of Social Sciences, Education and Social Work Ethics Committee, Queen’s University Belfast (Reference number 038_1819) for Samples 1 and 4 and from The School of Biological Sciences Ethics Committee, Queen’s University Belfast (0519/LavelleFA, 0519/LavelleFB), for Samples 2, 3 and 5.

## Results

### Overall usability, face validity, user feedback

The research team established initial face validity to ensure that all items measured what they claimed to measure. Piloting of the measures established that children could distinguish between ‘good’ and ‘poor’ performance of an illustrated skill and that they found the measure easy to use. Teacher feedback relating to the characters was positive and the teachers felt that the illustration would help the children struggling with literacy and/or would help children who had learning difficulties. However, it was noted that some children using the **CooC7** may still need help reading. Teachers recommended that a larger font size for **CooC11**, would be beneficial for the younger age group (8–9 years) to help with their reading. The children’s enjoyment completing the measure, especially using the measure on a tablet, and the short duration of time required to complete the measures were seen as positives.

Qualitative feedback showed that children completing the measure enjoyed doing it and wanted more questions and suggested that they should be given an opportunity to provide a reason why they don’t do certain skills. They also identified with the illustrated characters, *“That guy is just like me except the hair – like it’s me”* (P7 male pupil, sample 1).

### CooC11 (ages 8–12 years)

#### Exploratory factor analysis

The results showed an excellent KMO value of 0.86 and a significant (*p* < .001) Bartlett’s Test of Sphericity, indicating sample adequacy for analysis. Initially three factors were apparent in the data. ‘Tearing leaves,’ ‘Using a blender,’ and ‘Using a microwave’ were removed due to communalities < 0.25 and cross loading across factors. Given this the analysis was conducted again, to ensure that the factor structure and results were acceptable after the modification. Two factors were now apparent in the data, as shown by Eigenvalues greater than 1. Both factors had a minimum of 3 items, no items cross-loaded on more than one factor, and the minimum factor loading was 0.3. In addition, the internal reliability values for each factor were 0.77 and 0.72 respectively, therefore, all 11 items were retained. The overall Cronbach’s alpha for the measure was 0.82 in this sample.

#### Confirmatory factor analysis

**Fig. 3:**
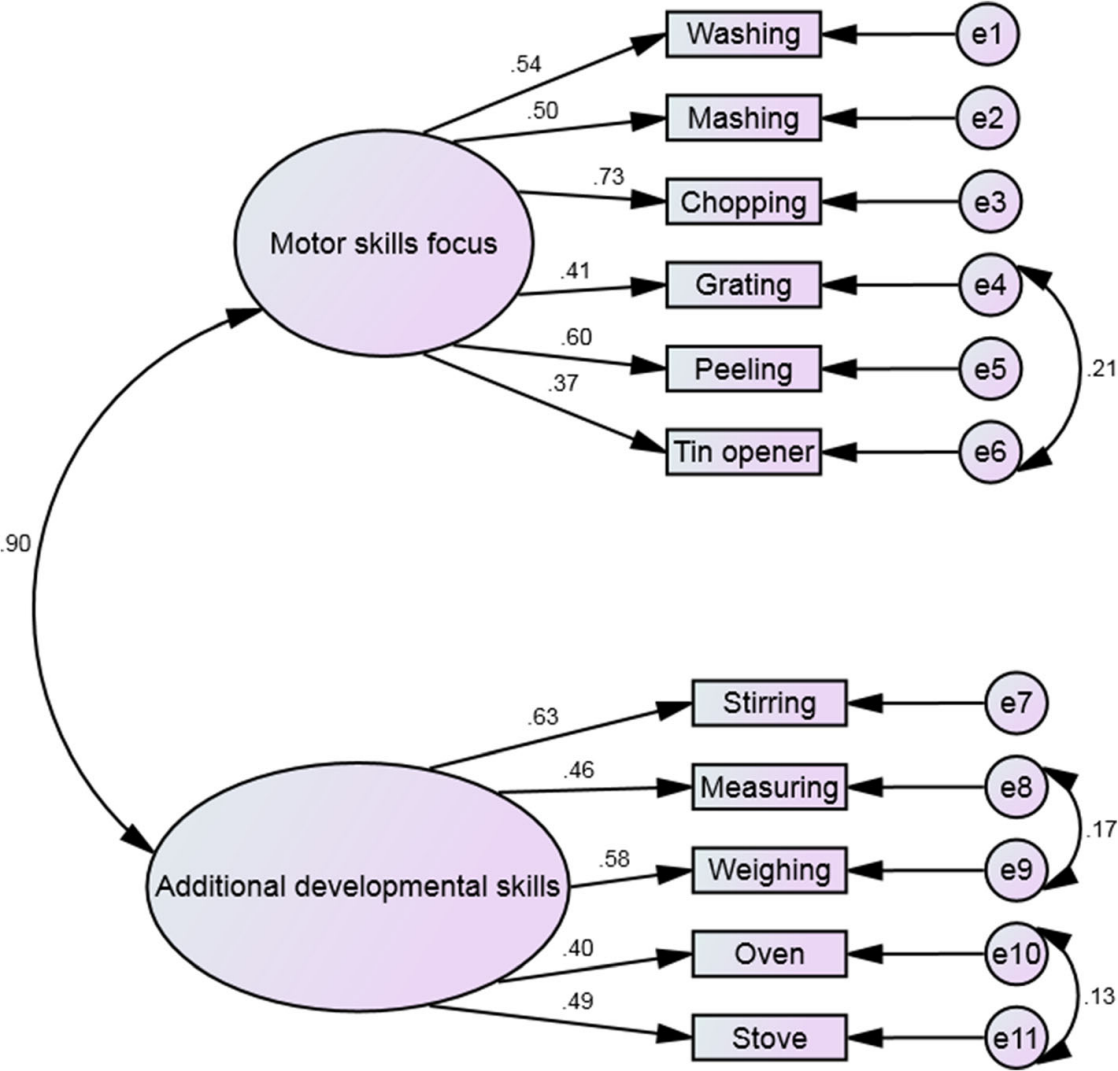
Final measurement model for CooC11 with standardised factor loadings and correlations

When entered as a CFA, the final EFA model did not have optimal fit (significant χ^2^, RMSEA 0.06, NFI 0.83, TLI .89). To improve this, the modification indices suggested some of the error terms should be allowed to covary. Following these amendments, fit was acceptable. Specifically, the χ^2^ was significant, but with a χ^2^/df ratio below 2 (1.50). RMSEA was 0.05. While the NFI was .87, the CFI was .95 and TLI .93, indicating overall acceptable fit. All standardised loadings were 30 or above (see Fig. 3 for final model). Face validity was established through the agreement that all items were appropriate for their factor, after discussion around ‘stirring.’ As ‘stirring’ may seem more appropriate to a motor skills focus, however, as highlighted in the original review, ‘stirring’ could be considered over heat, which has additional safety components. Due to it being considered an easier skill, it appeared before ‘using a hob/stove’ which was added to factor in this difference and therefore some children may have still considered it over heat, and thus agreement was reached it was appropriately placed.

#### Construct validity (convergent and discriminant validity)

In sample 1 (*N*=469), the adults cooking methods confidence score had a Cronbach’s Alpha of 0.82. Spearman’s rank correlation analyses showed that the children’s cooking competence measure in the current study was significantly correlated with the adults cooking methods confidence score, 0.49 (*P*< 0.001). Those children that reported helping their parents with preparing the dinner had a significantly higher cooking competence than those who did not (P< 0.001), see Table 2.

**Table 2:** Differentiating between those that report helping prepare dinner and those that don’t

Measure	Total Sample (***N*** = 272)	Do not help with dinner (***N***=136)	Help with dinner (***N***=136)	Significance
	M(SD)	M (SD)	M (SD)	P
**CooC11**	17.04 (12.88)	10.57 (9.65)	23.51 (12.47)	0.000

#### Internal consistency and temporal stability

The internal consistency reliability of **CooC11** was very good for the 8–9 years and 10–11 years, with a Cronbach’s of 0.86 and 0.84, respectively, and 0.85 overall.

In terms of temporal reliability **CooC11** had an ICC of 0.91, indicating an excellent temporal stability, with the two subscales having good temporal stability, as detailed in Table 3.

**Table 3:** Temporal Stability of CooC11 and factors

Scale	T1	T2	ICC
	*M (SD)*	*M (SD)*	
Motor skills focus	7.50 (8.04)	6.97 (7.35)	0.89
Additional Developmental skills	6.37 (6.44)	7.61 (7.11)	0.85
**CooC11**	**13.87 (13.42)**	**14.58 (13.16)**	**0.91**

#### Responsiveness to change

The measure is responsive to change, as seen by a significant increase (*P*< 0.01) from pre-cooking camp intervention **CooC11** mean (SD), 21.75 (7.89), to post camp CooC11, 26.13 (8.89).

### CooC7 (ages 6–7 years)

#### Exploratory factor analysis

The results showed an excellent KMO value for **CooC7** of 0.81 and a significant (p < .001) Bartlett’s Test of Sphericity, indicating that the sample was adequate for factor analysis. Two factors were seen in the data, as shown by Eigenvalues greater than 1. All factors had a minimum of 3 items. ‘Tearing leaves’ and ‘Stirring/mixing’ were removed due to communalities < 0.15. Furthermore, ‘Using a tin opener’ was removed as it did not meet the minimum factor loading of 0.3 and cross loaded across factors. Given this the analysis was conducted again to ensure that the factor structure and results were acceptable following the previous modification. All factors contained at least three items, no items cross-loaded on more than one factor, and the minimum factor loading was 0.3. In addition, the Cronbach’s Alpha values for each factor were 0.65 and 0.62, respectively, therefore, all 7 items were retained. The overall measure had an internal consistency reliability of 0.71.

#### Confirmatory factor analysis

**Fig. 4:**
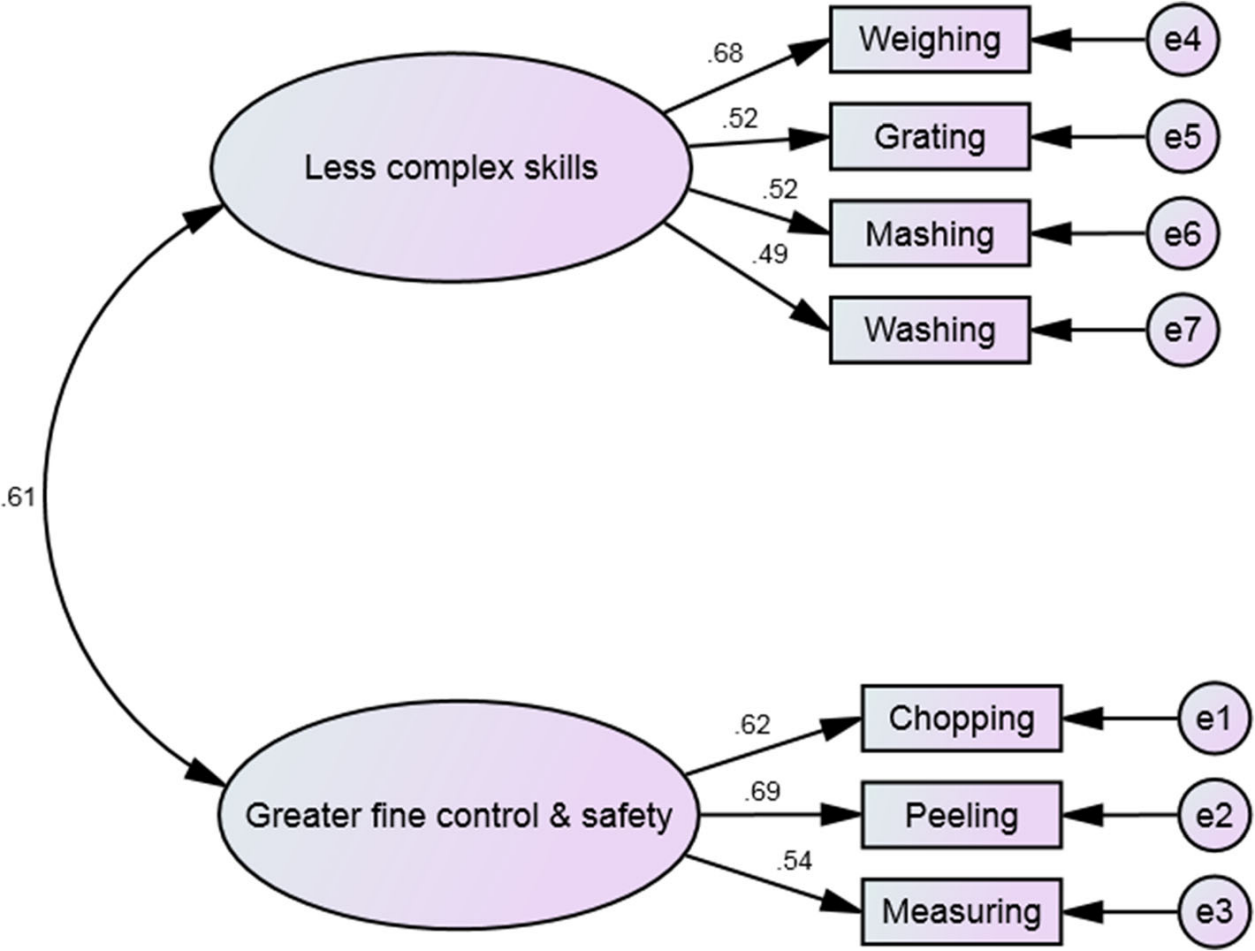
Final measurement model for CooC7 with standardised factor loadings and correlations

The final EFA model fit the data well in the confirmatory model (Fig. 4). The χ^2^ was non-significant, with a χ^2^/df ratio of 1.24, and the RMSEA was 0.04. The CFI, NFI, and TLI were all excellent at .98, .93, and .98 respectively. In addition, all standardised loadings were above 3. Face validity was established through the agreement that all items were appropriate for their factor after discussion around the factoring of weighing and measuring on separate factors.

#### Construct validity (discriminant validity)

Discriminant validity results for **CooC7** can be seen in Table 4 below.

**Table 4:** Differentiating between the different levels of assisting preparing dinner

Measure	Total Sample (***N***=513)	Never help with dinner (***N***=190)	Sometimes help with dinner (***N***=205)	Always Help with dinner (***N***=118)	Significance
	*M (SD)*	*M (SD)*	*M (SD)*	*M (SD)*	*P*
**CooC7**	11.83 (8.66)	8.12 (7.71)^a^	12.17 (8.17)^b^	17.20 (8.02)^c^	0.000

Superscript letters depict where significant differences (*P* < 0.001) fall between the groups.

#### Temporal stability

In terms of temporal reliability the measure had an ICC of 0.92, indicating an excellent temporal stability, with the two subscales having good temporal stability (see Table 5).

**Table 5:** Temporal Stability of measure and factors

Scale	T1	T2	ICC
	*M (SD)*	*M (SD)*	
Less Complex skills	7.00 (4.81)	7.62 (7.07)	0.85
Greater fine control & Safety	5.23 (5.00)	4.77 (4.89)	0.86
**CooC7**	**12.23 (9.00)**	**12.39 (10.14)**	**0.92**

## Discussion

To the best of our knowledge, this research is the first to develop and validate age appropriate children’s measures, **CooC11** and **CooC7,** for the design and evaluation of nutrition and cooking interventions. Effectively measuring perceived cooking competence is an important element for assessment of interventions, as perceived competence has been shown to be a motivator for repeating the behaviour [25] and cooking has been associated with a better diet quality [7, 8]. Both measures were developed to ensure they were age appropriate and engaging for children [31, 33]. They have high levels of face and construct validity and excellent internal consistency reliability and temporal stability. Additionally, **CooC11** demonstrated responsiveness to change validity.

For both measures endpoint user feedback was generally positive and children found completing the measures enjoyable. The children understood the language being used, enjoyed the character illustrations and use of a tablet to complete the measure, indicating an appropriate measure format, and requested additional questions, highlighting that completing the measure was within their attention span [31, 32].

### Factors

The EFA and CFA confirmed a two-factor structure for both **CooC11** and **CooC7**. For **CooC11**, face validity of the two factors was established, Factor 1 consists of cooking skills that encompass basic motor skills and Factor 2 consists of cooking skills that need additional developmental skills such as numeracy and literacy. Stirring was accepted in Factor 2, as this cooking skill is often performed over heat and therefore needed safety considerations, which was also noted in the original recommendations [39]. While the skill ‘using a stove/hob’ was added to encompass this type of stirring, e.g. stirring over heat, the skill ‘stirring’ is shown to the child before ‘using a stove/hob’ so they may still consider ‘stirring’ as over heat.

**CooC7**, also has two factors. Factor 1 consisted of the less complex skills for this age group whereas, Factor 2 consisted of cooking skills that required greater fine motor control and safety aspects. While there was some initial discussion around ‘grating’ factoring onto Factor 1, it was established that this cooking skill requires less fine motor control than ‘using a peeler’ or ‘chopping.’ Additionally, in this age group, ‘measuring liquids’ and ‘weighing’ factored onto different factors. It is suggested that for this age group, ‘measuring liquids’ is a more complex skill requiring greater fine motor control to accurately measure out liquids correctly.

While these factors are apparent within the measures, using **CooC11** and **CooC7** in their entirety is currently recommended, as these measure are quick to complete and there is a lack of validated measures to assess the factor construct validity.

### Validity and reliability

The **CooC11**, was correlated with an adult cooking confidence measure [40], which showed some convergent validity. Both measures were able to distinguish between children who reported different levels of assisting their parents with preparing the dinner, highlighting initial discriminant validity. Furthermore, **CooC11** showed it was responsive to change, as there was a significant increase in perceived cooking competence by children attending the cooking camp intervention. It is worth noting that due to the nature and recruitment for this intervention, these children had an initial interest in cooking, shown by their higher initial **CooC11** scores compared to the children in samples 1 and 2 who were recruited from schools. As changes were detected pre and post intervention, this demonstrates that the measures can be used with participants who have some level of initial cooking competence to measure changes.

Both measures showed that they had high levels of internal consistency reliability, indicating that they are measuring coherent concepts, demonstrated by the Cronbach’s alphas being greater than 0.7, which is satisfactory for non-clinical samples [54, 55]. Additionally, both overall measures showed excellent temporal stability demonstrated by the test-retest analysis, where the ICC scores were greater than 0.9 [52], showing that the scores are highly reliable over time.

### Strengthening the research area

The reported decline in child involvement in cooking in the home environment [56, 57], has led to an increase in children’s cooking interventions [7, 8]. New models are being created, such as the Cook-Ed™ model [58] to help guide the design, development and evaluation of the quality and success of the interventions using validated measures. The new **CooC11** and **CooC7** are a necessity in this area and will contribute to the strengthening of the global research by providing validated measurements to use in the evaluation of intervention studies. The measurement of perceived competence is a key element for consideration in the evaluation of interventions, as perceived competence has been shown to be a motivator for repeating the behaviour [25].

### Future research

Future research should assess the responsiveness to change of **CooC7.** Due to the difficulty in recruiting this age group within the available resources this was not assessed within the reported studies. Endpoint user feedback from teachers suggest that these new measures can be adapted to be used with people with learning difficulties and/or disabilities. Future research could develop and diversify the characters to make the measures suitable to use with these target populations.

### Strengths and limitations

The new measures involved extensive development, underpinned by a review, expert consultation and existing measures [29, 40] and illustrated by a graphic designer in consultation with a chef. The illustrated characters were highlighted as helpful and relatable. Additionally, the use of tablets to complete the measure was enjoyable to the participating children and the use of characters and a tablet to undertake the measures, enabled a more ‘game’ feeling rather than a test, which was found to be beneficial [33]. However, it was noted, to help with literacy for those at the younger end of the age range using **CooC11**, an increased font size would be beneficial.

The developed measures were found to be highly reliable and valid. The cooking skills included were based on evidence based age-appropriate recommendations developed from global publically available cooking recommendations and deconstructed for their underlying motor skills [39], thus increasing the generalisability of the measures outside of a UK/Irish population. Due to the lack of ethnic diversity in the regions being sampled, the children were shown Caucasian illustrations, however, to increase the cross-cultural applicability of the measures, a range of diverse character illustrations are available to use to ensure that all children can identify with the characters illustrated. While the measures assess specific skills and the illustrations only act as a ‘prompt’ for skill identification, some examination of the use of different characters in the measure may be of interest. Furthermore, if individual factors in the measures are to be used as standalone measures then construct validity of the factor structure is necessary when additional measures are available.

## Conclusions

The **CooC11** and **CooC7** are the first extensively developed and validated age-appropriate measures for assessing children in nutrition related interventions. The measures assess children’s perceived **Coo**king **C**ompetence for children aged 8–12 and 6–7 years respectively and can be used to evaluate the efficacy of children’s cooking intervention studies or school programmes.

## Data Availability

The datasets used and/or analysed during the current study are available from the corresponding author on reasonable request. The measures are freely available to use and can be obtained by contacting the corresponding author. Additionally, the measure has been translated into the Irish language which is also available from the corresponding author. W*here appropriate the corresponding author is willing to assist with back translation for other translations.*
